# Comprehensive eye examination: what does it mean?

**Published:** 2019-12-17

**Authors:** Saumya Yadav, Radhika Tandon

**Affiliations:** 1 Senior Resident, Ophthalmology, Dr Rajendra Prasad Centre for Ophthalmic Sciences, AIIMS, New Delhi, India.; 2 Professor, Ophthalmology, Dr Rajendra Prasad Centre for Ophthalmic Sciences, AIIMS, New Delhi, India.

**A routine comprehensive eye examination helps to screen for and diagnose common eye diseases.**

**Figure:**
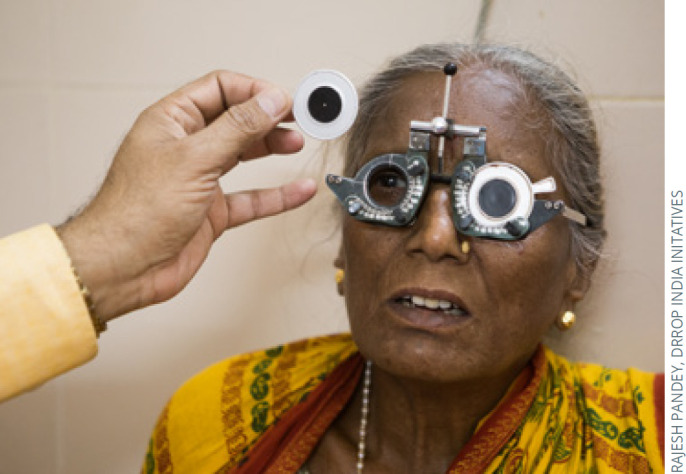
A patient getting checked for refraction. INDIA

As an eye health professional, it is important to talk to patients and the public about:

- consulting an ophthalmologist early when experiencing eye or vision-related problems and
- regular, periodic eye examination for early detection and treatment of eye diseases

In addition, it is a good practice to talk about basic measures for prevention of common eye diseases.^1–3^ A routine CEE presents a good opportunity to fulfil the above objectives. It helps to screen and diagnose common eye diseases, thus helping to reduce morbidity and the costs associated with eye diseases. A CEE is done when a patient with ocular symptoms seeks medical advice and also when a simple routine eye check-up is sought. This article explains what a CEE includes.

A CEE consists of a series of tests that assess the different aspects of eye health. Ideally, a CEE should be done not just for patients seeking medical advice but also for individuals above the age of 40 as a yearly check-up.

**Table 1:** Components of a comprehensive eye examination

Components	Tools
**External ocular examination**	Torch light
**Visual acuity test**	Snellen's chart Near vision charts
**Visual fields test∗**	Central 30–2 full threshold Humphrey visual field analyser Frequency doubling perimeter Goldmann kinetic perimeter
**Colour vision test∗**	Ishihara test
**Binocular vision∗**	Bagolini's striated glasses Worth four dot test Red filter test
**Stereopsis∗**	Random dot stereoacuity test TNO and Lang's stereo test
**Refraction**	Self- illuminated/mirror retinoscope Trial frame Set of trial lenses Cycloplegic drugs Jackson cross cylinder Automated refractometers
**Anterior segment and pupillary examination**	Torch light Slit lamp biomicroscope
**Gonioscopy∗**	Goniolens (Goldmann two, three and four mirror)
**Intraocular pressure**	Tonometer (Goldmann, Tono-pen, Perkins, Shiotz)
**Fundus evaluation**	Direct and indirect ophthalmoscope +90D/+78D lens
∗*Required if clinically indicated based on history and examination*	

In case, a routine annual review is not possible for the entire population, it should be recommended for those with:

- a known chronic eye disease
- a family history of glaucoma or
- a systemic disease known to affect the eyes such as diabetes mellitus

Do note, that these tests may vary depending upon the population examined and the infrastructure available at a clinic. (Table 1 lists various tests in a CEE).

## History

A detailed medical and treatment history is essential before beginning a CEE. Make a note of:

- family history of illnesses and working and living conditions to get an idea of the symptoms
- systemic illnesses like diabetes, hypertension, thyroid or inherited disorders. Such illnesses may affect the eyes and need appropriate investigations

**Figure 1:**
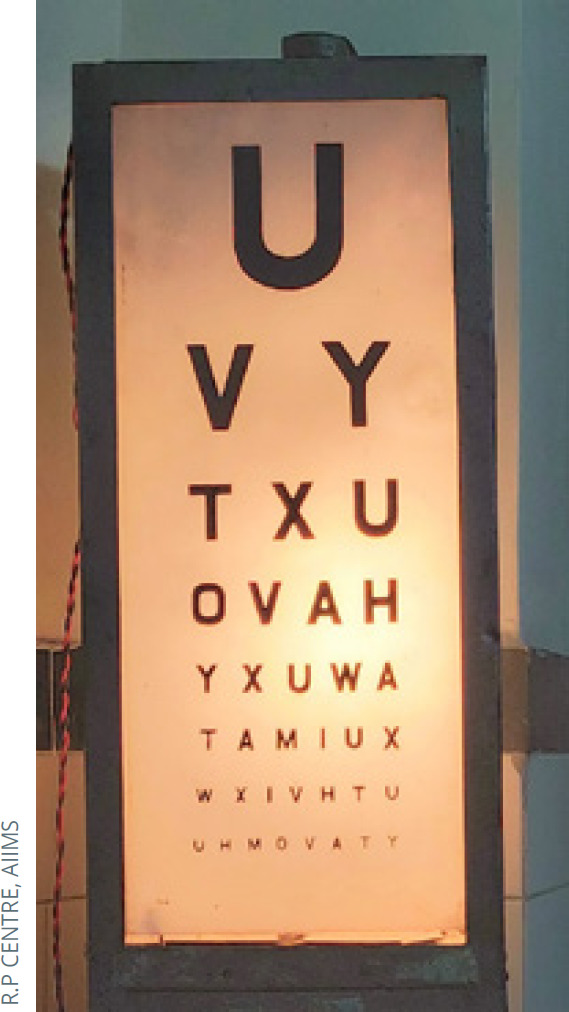
Snellen's visual acuity chart

**Visual acuity** (VA) is a measure of the eye's ability to distinguish shapes and the details of objects at a given distance. To measure VA, ask your patient to read letters on Snellen (Figure 1) or an E chart. Note the type of correction (spectacles/contact lenses) used by the patient. Any reduction in VA can show an underlying pathology. Write the results of the VA test as a fraction (20/40). The top number in the fraction is the standard distance at which a patient stands/ sits (20 feet). The bottom number is the smallest line of letter-size that the patient can read. Normal distance VA is 20/20. A pinhole test can distinguish if the reduced vision is due to refractive errors or other causes. Record the best corrected VA after you identify full correction of refractive error.

In young children, use Tellers and Cardiff acuity cards or optokinetic nystagmus. Measure the presenting and corrected near visual acuity with hand-held test cards by placing them at a distance of 40 cm.

**Visual field** can be tested using a simple procedure known as confrontation test. A confrontation test checks the peripheral and central visual fields (VF) and is the most used VF test done during a CEE. Each eye is tested for all four quadrants (upper and lower, temporal and nasal). In the confrontation test the eye examiner moves a target (usually a finger) from the periphery towards the centre and asks the patient when they see the target.

Perimetry tests are used for a more detailed and systematic evaluation of VF. Amsler grid is a useful tool for macular disorders with central field defect (age-related macular degeneration). Testing the visual field is useful in the management of patients with glaucoma, neuro-ophthalmic and retinal disorders.

**Contrast sensitivity** is the measure of the eye's ability to detect an object against its background. A Pelli Robson chart is used to test for contrast sensitivity. The Pelli Robson chart consists of horizontal lines of capital letters in contrast of one colour. Glaucoma, diabetic eye disease, and cataracts have shown to reduce contrast sensitivity in patients.

**Colour vision** deficiency is the inability to distinguish between certain shades of colour. It is a genetic disorder more common in men. Red-green deficiency is most common. Conditions like diabetes, glaucoma, optic neuritis and use of certain drugs (chlorpromazine, thioridazine, ethambutol) may lead to colour vision deficiencies. Many patients are unaware of their deficiency unless tested. We recommend use of colour vision charts for screening and detecting specific types of colour blindness.

**Binocular vision** is the vision achieved by the coordinated use of both eyes together. Simultaneous perception, fusion, and stereopsis are the three grades of binocular vision. Binocular vision can be tested using Bagolini's striated glasses, Worth four dot test and red filter test.

**Figure 2:**
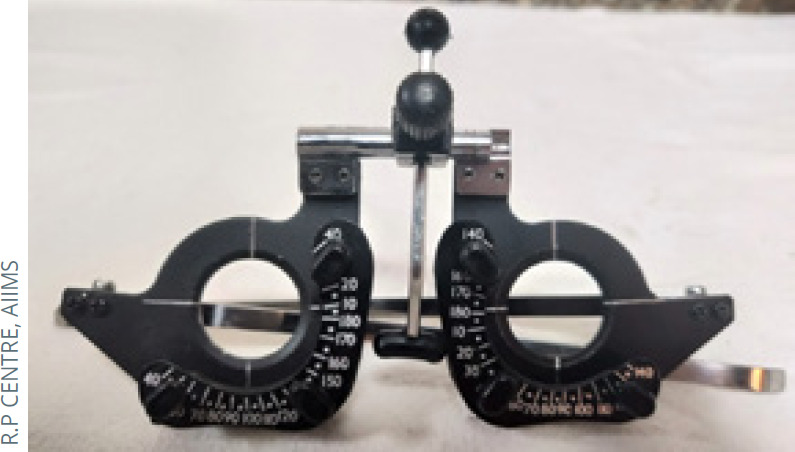
A trial frame. INDIA

**Refraction** is a test that determines the type (myopia, hypermetropia, and astigmatism) and the amount of refractive error (RE). It also tells us the required lens power needed to compensate for it. For a correct estimate of RE, the patient's accommodation should be minimal. Accommodation is the ability of the eye to change focus from distant to near images. Dry retinoscopy is the technique of refraction done without using cycloplegics. Here you can control the accommodation by asking the patient to fixate at a distant target. In wet retinoscopy, cycloplegic drugs are used to paralyse the ciliary body and remove the influence of accommodation during the test. Use a self-illuminated or mirror retinoscope to measure refractive error by placing a series of lenses in trial frames (Figure 2) in front of the eyes. You can also use automated refractometers for an initial estimate of RE. You can fine-tune your estimates using Jackson cross-cylinder and lenses to help the patient gain clearest vision.

We recommend cycloplegic refraction followed by a post- mydriatic test for adequate assessment of RE in infants and young children. For correction of presbyopia, we prescribe adding a plus lens over the patient's distance refractive correction.

**Figure 3:**
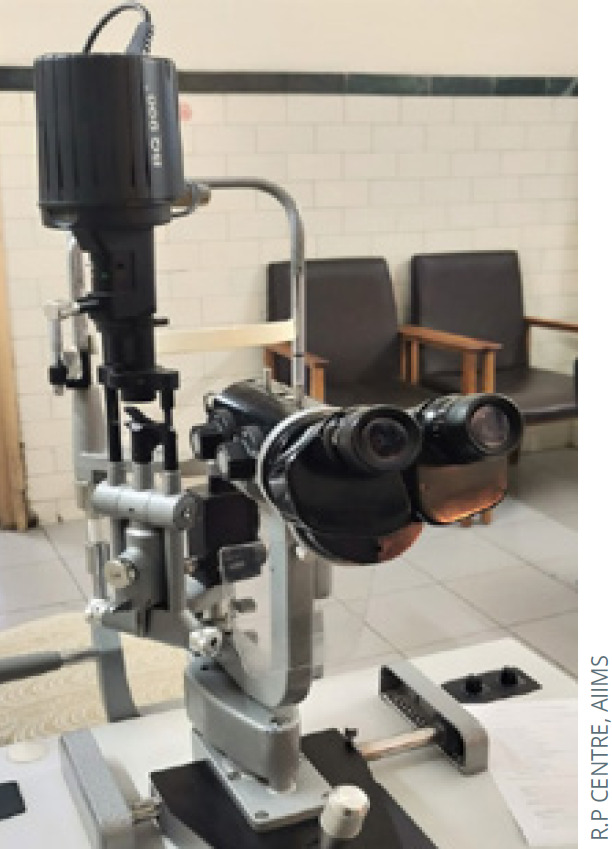
Slit- lamp biomicroscope. INDIA

### Torchlight external eye exam

An external torchlight examination helps to inspect:

- alignment and position of the eyes, eyelids, adnexa, conjunctiva, sclera, cornea, iris, pupils and extraocular movements
- palpebral symmetry, lid abnormalities, redness or growths on the conjunctiva and presence of any discharge (see Table 2)
- cornea for any abnormalities
- pupils for their size, shape, location, and reactivity

You can test the eye movements (versions and ductions) by asking the patient to look in nine cardinal positions of gaze. Use cover/uncover test to look for underlying heterophoria. Prism bar alternate cover test measures the total amount of deviation. In cases where prism bar can't measure the deviation, you can use Hirschberg and modified Krimsky tests.

### Slit-lamp biomicroscopy for anterior segment

A slit-lamp (Figure 3) examines the anterior and posterior segment of the eye, which includes conjunctiva, cornea, anterior chamber, pupil, lens and retrolental space (see Table 2). Gonioscopy is the technique of visualising anterior chamber angle structures at the SL. Findings from gonioscopy include the width of angle, presence of peripheral synechiae, goniosynechiae, hyperpigmentation, and neovascularisation.

### Intraocular pressure

Tonometry is used to measure intraocular pressures (IOP) and to evaluate patients with or at risk of glaucoma. Different types of tonometers include:

- applanation tonometry (Goldmann and Perkins applanation tonometry, non-contact tonometry, ocular response analyser)
- indentation tonometry (Schiotz tonometer, pneumotonometer, tono-pen)
- rebound tonometry
- Pascal dynamic contour tonometer

### Preliminary assessment of the posterior segment with distant direct ophthalmoscopy

Distant direct ophthalmoscopy (DDO) is performed routinely before a dilated fundus examination. DDO helps in diagnosing media opacities. Use a self-illuminating retinoscope or ophthalmoscope in a semi-dark room at a distance of 20–25 cm from the patient's eye. Note the features of red glow in the pupillary area. You may see abnormal greyish pupillary reflex in cases of cataract or some retinal detachments.

### Detailed fundus exam with a direct, and indirect ophthalmoscope and slit lamp biomicroscopy

Direct ophthalmoscopy provides an upright and monocular image of the retina. It is very useful for examining optic disc changes and foveal pathologies at higher magnification. A dilated fundus evaluation using a binocular indirect ophthalmoscope or SL biomicroscope with a +90Dioptres (D)/+78D lens is essential to record pathologies affecting the peripheral retina. Limited field of view is one limitation of direct ophthalmoscopy.

A dilated fundus examination helps to rule out diseases like diabetic retinopathy (DR) which have a high prevalence. Non-mydriatic fundus cameras are also available for peripheral centre-based screening of DR.

After a CEE, consider the results of the examination to determine a diagnosis. Sometimes more investigation may be needed to confirm or rule out the suspected diagnosis and to develop a treatment plan. Make appropriate referrals if your patient needs specialist consultations.

**Table 2:** Ocular structures and related disorders to look for during a comprehensive eye examination

Ocular structures	Disorders
**Eye brows**	Madarosis (Leprosy, Myxedema)
**Eye lids**	Ptosis Lid retraction Lagophthalmos Entropion Ectropion Trichiasis Distichiasis Blepharitis Chalazion Stye
**Palpebral aperture**	Blepharophimosis Ankyloblepharon
**Lacrimal apparatus**	Fistula Punctual stenosis Regurgitation
**Eye balls**	Proptosis Anophthalmos Enophthalmos Heterotropias
**Conjunctiva**	Discolouration Conjunctivitis Chemosis Circumcorneal congestion Pterygium Pinguecula Follicles Papillae Symblepharon Foreign body
**Sclera**	Discolouration Episcleritis Scleritis Staphyloma Perforations
**Cornea**	Microcornea Megalocornea Keratoconus Keratoglobus Cornea plana Dry Eyes Edema Scarring Degenerations Ulceration Vascularisation Guttae Keratic precipitates Keratitis
**Anterior chamber**	Shallow/irregular depth Aqueous cells/flare Hypopyon
**Iris**	Heterochromia Synechiae Iridodonesis Rubeosis iridis Transillumination defects
**Pupil**	Shape (festooned pupil) size (anisocoria, traumatic mydirasis), Colour (leucocoria, greyish reflex) RAPD (swinging torch light test) Correctopia
**Lens**	Dislocation Subluxation Cataract
**Optic disc**	Glaucoma Papilledema Papillitis Optic atrophy
**Macula**	Macular hole Haemorrhage Cherry red spot Oedema Hard and soft exudates ARMD
**Retinal vasculature**	Diabetic and hypertensive retinopathy CRVO CRAO Vasculitis
